# Ubiquitylation, neddylation and the DNA damage response

**DOI:** 10.1098/rsob.150018

**Published:** 2015-04-01

**Authors:** Jessica S. Brown, Stephen P. Jackson

**Affiliations:** The Wellcome Trust and Cancer Research UK Gurdon Institute, University of Cambridge, Cambridge CB2 1QN, UK

**Keywords:** DNA damage response, double-strand break repair, ubiquitin, NEDD8, MLN4924

## Abstract

Failure of accurate DNA damage sensing and repair mechanisms manifests as a variety of human diseases, including neurodegenerative disorders, immunodeficiency, infertility and cancer. The accuracy and efficiency of DNA damage detection and repair, collectively termed the DNA damage response (DDR), requires the recruitment and subsequent post-translational modification (PTM) of a complex network of proteins. Ubiquitin and the ubiquitin-like protein (UBL) SUMO have established roles in regulating the cellular response to DNA double-strand breaks (DSBs). A role for other UBLs, such as NEDD8, is also now emerging. This article provides an overview of the DDR, discusses our current understanding of the process and function of PTM by ubiquitin and NEDD8, and reviews the literature surrounding the role of ubiquitylation and neddylation in DNA repair processes, focusing particularly on DNA DSB repair.

## 2. Introduction

Organisms have developed elaborate cellular pathways that encompass the sensing, signalling and repair of damaged DNA, collectively termed the DNA damage response (DDR), in order to protect themselves from the long-term adverse effects of DNA damage [1,2]. The cascade of events that takes place following an insult to DNA involves the recruitment and localization of DNA damage sensor and mediator proteins into visible sub-nuclear foci [3]. Signalling pathways link these, often chromatin-associated complexes, with transducer/effector proteins that move throughout the nucleus, serving to amplify the DNA damage signal and to coordinate global as well as more localized cellular changes. The cellular response to DNA damage largely depends on the nature of the insult and subsequent type of damage, with DNA double-strand breaks (DSBs) being the most cytotoxic. The whole DDR process is tightly controlled by reversible protein post-translational modifications (PTMs), including phosphorylation, poly(ADP-ribosyl)ation, ubiquitylation, sumoylation, methylation, acetylation and others, which regulate protein stability, localization and activity without the need for changes in *de novo* protein synthesis.

DNA damage comes in many different forms, which may arise in isolation, or occur as a complex mixture depending on the nature of the insult. In addition, spontaneously arising DNA lesions contribute to mutagenesis and ageing [4]. DNA damage can result from endogenous sources, such as reactive oxygen species or other by-products of cellular metabolism, DNA mismatches during replication or as a result of abortive topoisomerase activity. DNA DSBs can also arise through programmed cellular events, such as during chromosomal crossover and recombination in meiosis or through V(D)J and class-switch recombination in developing lymphocytes to generate immune receptor and antibody diversity [5–7]. Alternatively, exogenous sources of DNA damage include ionizing radiation (IR), ultraviolet light (UV) and environmental carcinogens, including those derived from tobacco smoke.

Clinical syndromes arising due to hereditary defects in DDR proteins are typified by immunodeficiency, infertility, neurodegeneration, cancer predisposition and, in some cases, accelerated ageing, highlighting some of the physiological processes that rely on functional DNA repair pathways [1,2]. Genomic instability in particular is a hallmark of cancer, and many tumours are deficient in one or more DNA repair pathways. This, along with the inherent replication stress in many tumours, provides a therapeutic window for cytotoxic chemotherapeutics that act through the generation of DNA damage and has also led to the clinical development of small molecule inhibitors of key DDR enzymes [8–10].

### 2.1. Sensing a DNA double-strand break

A DSB is detected very quickly by various DSB ‘sensor’ proteins that subsequently direct signalling and repair via one of two predominant DSB repair pathways in human cells: homologous recombination (HR) or non-homologous end-joining (NHEJ). One of these DSB sensors is the Ku protein, a heterodimer formed by two structurally related polypeptides of 70 and 83 kDa (Ku70 and Ku80, respectively) [11,12]. Ku is a highly abundant DNA-binding protein, capable of binding free DNA ends, and is essential for repair by NHEJ [13,14]. DNA binding of Ku occurs rapidly following a DSB and is independent of DNA sequence [15–17]. Ku is able to self-associate and the binding of two Ku molecules to either side of the DSB enables bridging of Ku and stabilization of the DNA ends, while maintaining access to the DNA ends by ligation enzymes [18–20]. In addition, Ku serves to recruit all other core components of the NHEJ complex, including DNA-PKcs [17,21,22], XRCC4/LIG4 [23,24], XLF [25] and the recently identified PAXX protein [26,27], to enable DNA end-ligation/repair.

Another DSB sensor is the (MRN) protein complex comprising MRE11 (meiotic recombination 11), RAD50 and NBS1 (Nijmegen breakage syndrome 1) [28–31]. MRE11 has intrinsic DNA-binding activity [32], as well as endo- and exonuclease activity [33,34]. It is important for the short-range stabilization of DNA ends and, together with its binding partner CtIP (also known as RBBP8; retinoblastoma binding protein 8), promotes initiation of DNA end resection to promote HR [35,36]. The MRE11–RAD50 components of MRN also partially unwind DNA ends and are believed to play a role in the long-range tethering of DNA molecules, whereas NBS1 contributes to recruitment and activation of ATM (ataxia-telangiectasia mutated) kinase, which mediates downstream signalling events [37–40].

The poly(ADP-ribose) polymerase proteins PARP1 and PARP2 also recognize both single- and double-stranded DNA lesions, with such binding triggering their enzymatic activities to synthesize poly (ADP)-ribose (PAR) chains attached to PARP1/2 themselves as well as other proteins in the vicinity of DNA breaks [41–43]. The best-described DDR function for PARP is in single-strand break (SSB) repair, where PAR chains promote recruitment of DNA repair factors such as XRCC1 (X-ray repair cross-complementing protein 1) and LIG3 (DNA ligase 3) [44,45]. PARP1 also promotes DSB repair by alternative NHEJ [46]. It is not yet clear how a particular DSB might promote the recruitment of one over another DNA damage sensing molecule and, indeed, studies have shown that both MRN and Ku co-localize at some DSBs, at least initially [16]. DSB repair pathway choice at various levels of the signalling cascade is an area of intense current research, with recent work highlighting how it is at least in part controlled by cell cycle status [47] and chromatin structure [48].

### 2.2. Signalling events following a double-strand break

Even a single DSB can evoke a complex cellular response that occurs not only in the vicinity of the break but also globally throughout the cell to coordinate the most appropriate outcomes. DNA DSB signalling events are largely coordinated by the apical phosphatidylinositol 3-kinase-related kinases (PIKKs): ATM, ATR (ataxia-telangiectasia and Rad3-related protein) and DNA-PKcs. These kinases preferentially phosphorylate serine or threonine residues followed by a glutamine residue (S/TQ) [49]. Hundreds of potential substrates have been identified for ATM and ATR [50], although the physiological relevance of many of these is still not known. Interestingly, DNA-PKcs itself is the only physiologically relevant DNA-PK substrate identified to date [51,52]. As described above, ATM is predominantly activated following DSB formation by its interaction with NBS1 [37–39], although ATM activation can be potentiated by other factors, particularly in the context of a damaged or disrupted chromatin state [53]. ATR, however, is activated by RPA (replication protein A)-bound single-stranded DNA (ssDNA), which can arise either as a result of replication stress (following uncoupling of the replication helicase and polymerase) or following DNA end resection, as associated with HR-mediated DSB repair [54]. ATR is recruited to RPA-ssDNA by its obligate partner ATRIP (ATR-interacting protein), where it is activated by TOPBP1 (topoisomerase binding partner 1) [55–57]. Germline mutations in ATM or ATR result in ataxia-telangiectasia [58,59] and Seckel syndrome, respectively [60,61]. The catalytic activity of DNA-PK is brought about via Ku-mediated DNA binding [21] and promotes NHEJ [62–64]. Germline mutations in DNA-PKcs result in severe combined immunodeficiency syndromes [65].

**Figure 1.**
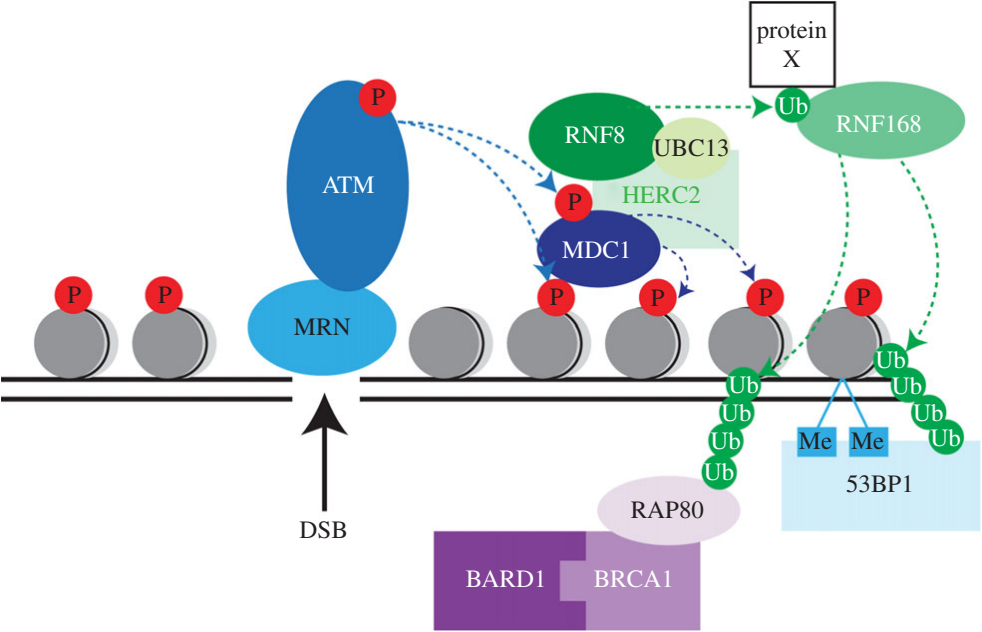
Simplified illustration of the major protein players involved in ubiquitin signalling following DSB induction. See text for details. Horizontal lines represent DNA. P, phosphorylation; Ub, ubiquitylation; Me, methylation. Protein X denotes unknown protein.

A critical early step in the cellular response to DNA DSBs is phosphorylation of histone H2AX on serine 139 (known as γH2AX) [66], largely by ATM in response to IR although functional redundancy exists with ATR and DNA-PK [67]. MDC1 (mediator of DNA damage checkpoint protein 1) directly binds γH2AX through its carboxyl-terminal BRCT repeats and potentiates the γH2AX signal, by both promoting its phosphorylation and curtailing its dephosphorylation [68]. Spreading of γH2AX to over a megabase from the site of the initial lesion [66] is required to effectively sustain the DNA damage signal sufficiently to recruit and retain mediator proteins such as 53BP1 at IR-induced foci. MDC1 also serves as a docking site for recruitment of other DDR proteins on the damaged chromatin and is the cornerstone molecule for crosstalk between phosphorylation and ubiquitylation signalling cascades in the DDR (figure 1).

As well as coordinating the local recruitment of mediator proteins to DNA DSBs, the PIKKs also phosphorylate effector molecules that regulate more global cellular responses, including transcription, apoptosis, senescence and delayed cell cycle progression. CHK1 (checkpoint kinase 1) and CHK2 (checkpoint kinase 2) are two well-characterized substrates of ATR and ATM, respectively, that function throughout the nucleus [69]. CHK1 is important for activation and maintenance of the G2/M checkpoint, whereas CHK2 is considered to work mainly, but not exclusively, in the G1/S checkpoint [70]. CDC25A is a principal substrate of CHK1, phosphorylation of which leads to SCF^βTRCP^-mediated degradation and activation of the intra-S and G2/M checkpoints [71]. A critical substrate of CHK2 is p53, phosphorylation of which promotes stabilization and subsequent apoptosis when the number of DNA lesions exceeds the repair capacity of the cell [72,73]. A third checkpoint kinase, MAPKAP-K2 (MK2), has also been characterized, which is the effector kinase of the p38 SAPK pathway [74]. In response to DSBs, the p38MAPK/MK2 pathway is activated downstream of ATM/ATR and functions independently of CHK1 activation to maintain G2/M and intra-S phase arrest [75]. Major substrates of MK2 following DNA damage include CDC25 family members as well as protein complexes important for RNA biology [74–76]. Together, the signalling pathways described above are critical for cell survival following DNA damage as they regulate the kinetics of cell cycle progression, allowing sufficient time for DNA repair.

### 2.3. DNA repair pathways

As mentioned previously, in human cells, the predominant DSB repair pathways are classical NHEJ and HR. NHEJ is initiated by the binding of Ku to DNA ends, which subsequently recruits DNA-PKcs, end-processing and ligation factors to the break. It is a relatively rapid repair pathway that occurs in all cell cycle stages. In the absence of classical NHEJ proteins, DNA DSBs can still be ligated, albeit more slowly, by an alternative NHEJ pathway (alt-NHEJ; also known as MMEJ—microhomology mediated end-joining) [77]. While it is generally assumed that alt-NHEJ only plays a detectable role in DSB repair when classical NHEJ is compromised, it appears to function more predominantly in the repair of certain types of breaks, such as those generated during immunoglobulin gene class-switch recombination [78]. Alt-NHEJ is thought to contribute to the excessive genomic deletions and chromosomal translocations seen in various tumours. PARP1, XRCC1, LIG3, LIG1 and CtIP have all been shown to play a role in alt-NHEJ; however, the exact mechanism of repair has not been fully defined [79].

In contrast to NHEJ, DSB repair by HR requires significant DNA end-processing and is initiated following 3′–5′ DNA end resection coordinated by CtIP and the MRN complex [2]. The resulting ssDNA is stabilized through RPA (replication protein A) coating, leading to ATR activation. BRCA2 (breast cancer type 2 susceptibility protein), probably with the help of BRCA1 (breast cancer type 1 susceptibility protein) and PALB2 (partner and localizer of BRCA2), promotes the loading of RAD51 onto RPA-coated ssDNA, which then enables strand invasion of a homologous DNA sequence in a sister chromatid and formation of a complex DNA arrangement that is resolved by mechanisms that may or may not result in crossover of the two DNA molecules [80]. HR is restricted to S/G2 phases of the cell cycle and, compared with NHEJ, is a relatively slow process, sometimes taking hours to complete. Hereditary defects in genes for HR factors such as BRCA1 and BRCA2 lead to cancer predisposition syndromes and tumours that are reliant on alternative repair pathways for survival. This observation led to the idea of using the synthetic lethality concept to treat such cancers and the clinical development of PARP inhibitors for the treatment of BRCA1- and BRCA2-deficient tumours [81–83]. Inhibitors of PARP activity with molecules such as olaparib/Lynparza^TM^ (AstraZeneca) inhibit SSB repair and also cause PARP to become trapped on DNA repair intermediates, resulting in DNA replication associated DSBs [84]. PARP inhibitors therefore generate one-ended DNA DSBs in cells that are normally repaired by HR processes; consequently, PARP inhibitors selectively kill HR deficient tumours [81,82].

Besides DNA DSBs, DNA damage can also consist of DNA SSBs, damaged DNA bases, DNA mismatches, as well as inter- and intrastrand DNA cross-links, which engage with specialized cellular pathways for their repair (table 1).

**Table 1.** Brief description of DNA repair pathways in human cells. See text for details on repair by NR and NHEJ.

DNA repair pathways	
mismatch repair (MMR)	DNA mismatches can arise during normal DNA replication and are repaired through MMR pathways involving the collective actions of a nuclease, polymerase and ligase [85]. Hereditary defects in MMR genes, such as occur in Lynch syndrome (also known as HNPCC, hereditary non-polyposis colorectal cancer) result in tumours with high levels of microsatellite instability
SSB repair	SSBs are recognized by PARP, which synthesizes PAR chains in the vicinity of the DNA break and promotes recruitment of DNA repair factors such as XRCC1 and LIG3 [86]. SSBs can occur as a result of IR or treatment with various chemical agents, and also arise as intermediates during BER and NER (see below)
base excision repair (BER)	involves the recognition, excision and replacement of damaged bases in cells, using enzymes that overlap with those required for SSB repair [87]
nucleotide excision repair (NER)	NER removes helix-distorting lesions from DNA, in particular the UV-induced photo lesions CPD (cyclobutane pyrimidine dimers) and 6-4PP (pyrimidine 6-4 pyrimidone photoproducts). Xeroderma pigmentosum (XP) is the archetypal human NER-deficiency syndrome, causing extreme sensitivity to UV light and very high incidences of skin malignancies. NER involves removal of a short oligonucleotide that includes the damaged lesion and subsequent restoration of the DNA sequence using the undamaged DNA as a template. Two sub-pathways of NER, global genome NER (GG-NER) and transcription coupled NER (TC-NER) use different mechanisms to recognize DNA lesions and promote either repair of DNA lesions throughout the genome or lesions encountered during active transcription, respectively [88]
trans-lesion synthesis (TLS)	TLS is a DNA damage bypass mechanism that protects against DSB break generation following replication fork stalling. It employs specialized DNA polymerases, principally from the Y-family, to replicate past the damaged DNA template and is inherently error-prone [89]
DNA interstrand cross-link (ICL) repair	ICLs can arise following exposure to a range of environmental mutagens, but are particularly abundant in cells following exposure to alkylating or platinum-based chemotherapeutics [90]. Fanconi anaemia is a rare genetic disorder causing aplastic anaemia, developmental defects and cancer predisposition, which is characterized by hypersensitivity to DNA interstrand cross-linking agents. It is caused by autosomal recessive mutations in one of 15 known genes that are required for ICL repair. The core Fanconi anaemia complex is made up of eight proteins (FANCA, B, C, E, F, G, L and M) required for the detection and repair of ICLs. ICLs can stall progression of the replication fork, causing replication fork collapse and the generation of a DNA DSB requiring coordination between translesion synthesis and homologous recombination mechanisms for repair [89]

## 3. Post-translational modification with ubiquitin

### 3.1. Ubiquitin and ubiquitin conjugation to substrates

**Figure 2.**
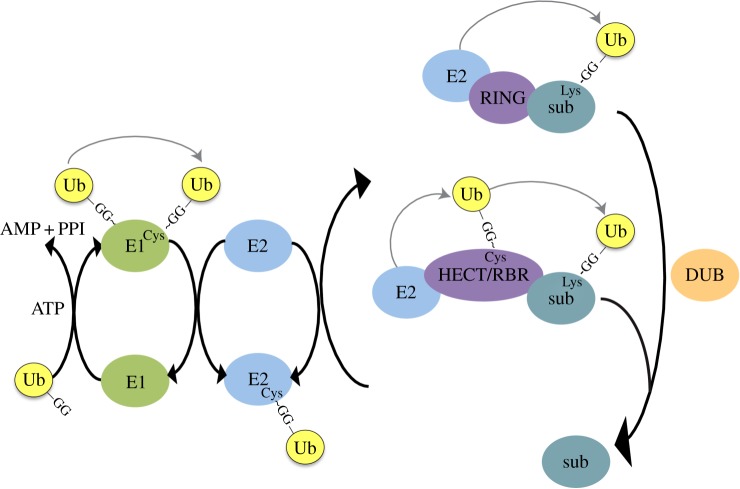
Illustration of ubiquitylation cascade. Ubiquitin is produced as a precursor polypeptide and cleaved to reveal a carboxyl-terminal GG- motif. In an ATP-dependent reaction, an E1 enzyme transforms this motif into a ubiquitin-adenylate intermediate, which reacts with a Cys in the catalytic domain of the E1 to form an E1∼Ub, thioester linkage. At least for UBA1 (the best-characterized ubiquitin E1), a second ubiquitin molecule is adenylated and remains non-covalently linked to the E1 adenylation active site. Double loading of the E1 with ubiquitin is believed to potentiate transfer of ubiquitin from the E1 to the E2 [99]. The ubiquitin-charged E1 is recognized by an E2 conjugating enzyme and ubiquitin is transferred to the catalytic cysteine of the E2 via a thioester linkage. Ubiquitin is subsequently conjugated to a substrate lysine, through E2 recognition of a substrate/E3 ligase complex. E1 and E3 binding sites to the E2 overlap, ensuring progression of the ubiquitylation cascade. RING E3s facilitate transfer of ubiquitin from the E2 to substrate without binding ubiquitin directly. Alternatively, ubiquitin is transferred to an active site cysteine in HECT/RBR E3s before forming an isopeptide linkage with the substrate lysine. Multiple cycles of substrate binding to ubiquitin-charged E2s lead to ubiquitin chain formation. Ubiquitylation can be reversed by de-ubiquitylating enzymes (DUBs).

Ubiquitin is a highly evolutionarily conserved, small (76 amino acid residue) protein, originally identified through its ability to mediate ATP-dependent protein degradation in reticulocyte extracts [91–93]. Four genes encode ubiquitin in the human genome (*UBC, UBB, UBA52* and *UBA80*), which are first transcribed either fused to ribosomal proteins (*UBA52, UBA80*), or as linear poly-ubiquitin chains that require processing to ubiquitin monomers (*UBC, UBB*) [94–96]. Full-length ubiquitin is a precursor peptide, requiring cleavage to expose a carboxyl-terminal di-glycine motif. Ubiquitin is then covalently conjugated via its carboxyl-terminus to target proteins, generally to the ε-amino group on a substrate lysine. This conjugation involves a three-step enzymatic process, first described in the 1980s [97,98], using an E1- (activating), E2- (conjugating) and E3- (ligase) enzyme (subsequently referred to as E1, E2 and E3, respectively; see figure 2 for further description of the enzymatic cascade).

A pyramid of enzymatic complexity exists to enable conjugation of ubiquitin to a plethora of substrates and the subsequent regulation of a wide range of biological processes. In humans, there are eight known E1s, two of which are specific for ubiquitin (UBA1 and UBA6) [100], 35 active E2s [101] and there are predicted to be more than 1000 E3s [102]. E3s can be divided into three major families: RING (really interesting new gene), HECT (homology to E6AP carboxyl-terminus) and RBR (ring between ring) [102,103]. The RING E3s bind simultaneously to the ubiquitin-charged E2 and substrate (either directly, or through E3-binding partners), facilitating the transfer of ubiquitin to the substrate, without the E3 binding ubiquitin directly. The RING domain of such E3s contains seven highly conserved cysteine residues and a highly conserved histidine residue that coordinate binding to two central Zn^2+^ ions, the structure of which is essential for E2 binding [104]. The term RING E3 ligase is rather a misnomer therefore, as RING E3s contain no catalytic activity *per se*, although they do catalyse the transfer of ubiquitin from the E2 to substrate by positioning the ubiquitin moiety into a favourable position for conjugation [105]. In the case of HECT and RBR E3s, however, ubiquitin is transferred from the E2 to an active site cysteine in the E3 and then to the substrate. U-box E3s are a smaller family of E3 enzymes, originally called E4s, as they were shown to fine-tune pre-formed ubiquitin chains [106]. They contain a U-box motif that has a similar three-dimensional structure to the RING domain but lacks the conserved Zn^2+^ binding residues; U-box containing proteins have subsequently been shown to have independent E3 ligase activity [107].

Unlike sumoylation and perhaps also neddylation, there is no target consensus motif for ubiquitin conjugation of substrates. In the majority of cases (especially for polyubiquitylation with K48 and K11 chains), ubiquitylation is not restricted to a particular substrate lysine, although examples of lysine-specific ubiquitylation do exist, for example K164 mono-ubiquitylation on PCNA (proliferating cell nuclear antigen). Depending on the E3 and substrate involved, lysine specificity can be determined by the E3, interacting partners of the E3 and/or the substrate itself [103].

Like all PTMs, ubiquitylation is dynamic and can be reversed by deubiquitylating enzymes (DUBs). There are five families of DUBs, comprising a total of 79 genes in the human genome (UCHs, ubiquitin carboxyl-terminal hydrolases; USPs, ubiquitin-specific proteases; OTUs, ovarian tumour proteases; MJDs, Machado-Josephin domain proteases; and JAMMs, JAB1/MPN/MOV34 metalloenzymes, also known as MPN+). Broadly speaking, the function of DUBs can be divided into three groups: processing of linear ubiquitin chains to generate free ubiquitin, editing of ubiquitin chains and reversal of ubiquitylation on substrates [108,109]. DUBs are generally highly specific for ubiquitin versus other UBLs, peptide versus isopeptide linkages, linkage chain type and different substrates. In addition, some DUBs have a preference for distal rather than proximal ubiquitin molecules, some preferentially cleave mono-ubiquitin and others have specially evolved for the recycling of ubiquitin following proteasomal degradation [108,109]. The balance between ubiquitylating and deubiquitylating activity is essential for regulating a wide range of cellular processes, encompassing all those described for ubiquitylation below.

### 3.2. Function of ubiquitylation and the ‘ubiquitin code’

Some substrates are modified by a single ubiquitin moiety on a single residue, termed mono-ubiquitylation, while others are multi-mono-ubiquitylated. Mono-ubiquitylation has been shown to regulate lysosomal degradation of proteins [110] and also mediates protein–protein interactions, such as regulating the recruitment of translesion synthesis polymerases following mono-ubiquitylation of PCNA after DNA damage [111]. Ubiquitin, however, contains seven lysine residues (K6, K11, K27, K29, K33, K48, K63), enabling the formation of ubiquitin chains and thus the poly-ubiquitylation of substrates. In addition, linear ubiquitin chains (also known as M1 linkage) can be formed using the amino-terminal methionine of ubiquitin [112]. The linkage specificity is largely determined by the pairing of specific E2s and E3s [113]. K48 poly-ubiquitylation was the first described, and labels proteins for proteasome-mediated degradation [114]. With the exception of K63-linked chains, all other chains accumulate following proteasomal inhibition in yeast [115], suggesting at least some role in directing proteasomal degradation for all non-K63-linked chains. Like mono-ubiquitylation, mediating non-proteasomal protein–protein interactions is an important additional function for poly-ubiquitylation, particularly K63 chains, and a number of different ubiquitin-binding domains (UBDs) have been characterized on a wide range of proteins [116,117]. The non-degradative K63 chains are particularly important in regulating the DDR and are discussed in more detail below. Other described functions for poly-ubiquitylation include the regulation of protein activity and localization [118,119]. Although mixed and branched ubiquitin chains are readily formed *in vitro,* the prevalence and physiological relevance of such linkages *in vivo* is still not fully clear [118]. Given the heterogeneity of the ubiquitin code, it is perhaps not surprising that ubiquitylation regulates a wide range of biological processes, including cell cycle progression, transcription, apoptosis and inflammation, as well as DNA damage signalling and repair. As such, its function or dysfunction is associated with various diseases, especially neurodegenerative disorders, viral diseases and cancer, and targeting ubiquitin-system components is currently an active area for drug development.

### 3.3. Ubiquitin-like proteins

Since the discovery of ubiquitin, a number of UBLs have been described, based on their structural similarity and sequence homology to ubiquitin. As discussed in more detail elsewhere, excluding ubiquitin itself, nine UBL families have been defined: NEDD8 (neural precursor cell expressed developmentally downregulated protein 8), SUMO1–3 (small ubiquitin-related modifier-1, -2 or -3), ISG15 (interferon-stimulated gene 15), URM1 (ubiquitin-related modifier 1), UFM1 (ubiquitin-fold modifier 1), FAT1 (HLA-F adjacent transcript 10), FAU (Finkel-Biskis-Reilly murine sarcoma virus ubiquitously expressed, also known as FUB1 or MNSF-β), ATG12 (autophagy-related protein 12), ATG8 (autophagy-related protein 8) and paralogues MAP1LC3 (microtubule-associated protein 1 light chain 3)-A, -B and -C and GABARAPL (gamma-aminobutyric acid receptor-associated protein-like)-1, -2 and -3 [100,120,121]. Like ubiquitin, the UBLs rely on an E1–E2–E3 enzymatic cascade to mediate their conjugation to substrates, although the enzymes used and downstream effects of conjugation are specific to each UBL. Along with SUMO, NEDD8 is one of the best-characterized UBLs to date, and the specifics of the NEDD8 conjugation pathway along with published functions of neddylation in the DDR are discussed further in following sections.

## 4. Role of ubiquitylation in DNA double-strand break repair

A role for ubiquitylation in the DDR first came through the discovery that the post-replication repair protein yeast Rad6 is actually a ubiquitin E2 [122] which, in association with the E3 Rad18, mono-ubiquitylates the replication factor PCNA on lysine 164 in response to DNA damage [123]. Subsequently, mono-ubiquitylation of PCNA by human RAD18 was shown to regulate the switch from the use of replicative polymerases to damage-tolerant polymerases at the replication fork [124,125]. Notably, in vertebrate cells RAD18-independent ubiquitylation of PCNA K164 also occurs, suggesting some differences between the regulation of post-replication repair in yeast and higher eukaryotes [126]. Mono-, as well as poly-ubiquitylation of PCNA is therefore crucial for coordinating DNA damage tolerance events in both yeast and human cells [89]. Ubiquitylation has since been shown to regulate almost all DNA repair pathways and, in particular, is integral to the early signalling events following DNA DSBs (figure 1).

### 4.1. Double-strand break signalling by RNF8 and RNF168

DNA damage sites are enriched with K63 ubiquitin chains [127,128], and RNF8 provides a critical link between phosphorylation and ubiquitylation events in the DDR (figure 1). Three independent studies in 2007 identified the RING ubiquitin E3 ligase RNF8 as a predominant E3 regulating ubiquitylation at DSB sites [129–131]. These studies demonstrated that RNF8 binds ATM-phosphorylated MDC1 via its FHA (forkhead-associated) domain, mediates K63-linked ubiquitylation at DNA damage sites with the E2 UBE2N (also known as UBC13) and is required for the recruitment of both 53BP1 and the RAP80/BRCA1 complex to DSB sites. In addition to binding phosphorylated MDC1, the FHA domain of mammalian RNF8 was also shown to interact with ATM-phosphorylated HERC2 (also a RING E3 ligase) following DNA damage to form an MDC1–RNF8–HERC2 complex [132]. Although it is not yet known whether the E3 ligase activity of HERC2 is required for its function in the DDR, in human cells, HERC2 has been reported to stabilize the interaction between RNF8 and UBE2N and also maintains the level of RNF8, to promote RNF8-mediated ubiquitylation and subsequent downstream events at DNA damage sites [132]. Notably, however, knockout of the gene for HERC2 in the chicken DT40 cell line does not cause DNA damage hypersensitivity or defects in ubiquitin accumulation at DNA damage sites [133], suggesting that at least some functions for this very large protein are not conserved throughout vertebrates.

The ubiquitylation signal required for DDR signalling is not sufficiently maintained by RNF8 alone, but is highly dependent on the activity of a second RING E3 ubiquitin ligase, RNF168. RNF168 MIUs (motif interacting with ubiquitin domains) and UMI (UIM and MIU-related ubiquitin-binding domain) bind as-yet undefined, RNF8-ubiquitylated substrates flanking DSB sites [127,128,134]. RNF168 then promotes ubiquitylation of histone H2A K13/15, thereby promoting assembly of 53BP1 and BRCA1/RAP80 complexes at sites of DNA damage [135]. Recent work has shed light on how a hierarchy of ubiquitin E3 recruitment is achieved in the DDR and has demonstrated that regions outside ubiquitin-binding domains, named LR motifs, as well as properties of the substrates themselves determine recruitment and substrate specificity of the E3 [136,137]. Current models propose that RNF8 ubiquitylates an as-yet unidentified non-nucleosomal substrate that serves as a docking site for RNF168 recruitment and subsequent ubiquitylation of lysines 13/15 on histone H2A(X), enabling recruitment of 53BP1 and BRCA1 complexes to DNA damage sites [135].

Functionally, bi-allelic heterozygous nonsense mutations in the gene encoding RNF168 cause RIDDLE syndrome (radiosensitivity, immunodeficiency, dysmorphic features and learning difficulties) [127,138] and depletion of either RNF8 or RNF168 in cells causes hypersensitivity to DSB inducing agents. Other studies have also demonstrated a role for RNF8/168 in immunoglobulin class-switch recombination [139], telomere end-protection [140,141] and transcriptional repression at DNA damage sites [142]. Interestingly, the DDR is suppressed during mitosis at the level of RNF8 function [143,144], highlighting the importance of RNF8-mediated ubiquitylation in driving DSB repair and associated signalling events.

### 4.2. Ubiquitylation impacts on 53BP1 and BRCA1 functions

The recruitment of 53BP1 and BRCA1 to DSB sites is essentially triggered by the same signal: RNF8/168-mediated ubiquitylation (figure 1). However, 53BP1 and BRCA1 have seemingly opposing functions, with 53BP1 promoting NHEJ [145] and BRCA1 promoting HR [146]. Until recently, despite being dependent on histone H2A K13/15 ubiquitylation, the recruitment of 53BP1 to DNA damage sites appeared to be through its TUDOR domain binding of histone H4K20me2 [147]. The demonstration that 53BP1 also contains a ubiquitylation-dependent recruitment (UDR) motif, required for its interaction with histone H2A ubiquitylated on K15 (H2A K15ub), has now explained the RNF168 dependency of 53BP1 recruitment to sites flanking DNA DSBs [148], with the current model proposing that 53BP1 binds nucleosomes bivalently, through interacting with both H4K20me2 and H2A K15ub at DNA damage sites [148].

The seminal studies on RNF8 demonstrated that the maintenance of BRCA1 at DSB sites depends on RAP80 binding of the BRCA1-A complex, via its ubiquitin-binding modules (UIMs), to K63-linked ubiquitin chains on chromatin [129–131]. Early recruitment of BRCA1 to damaged sites occurs independently of RAP80 however [149], and may be explained by the direct binding of BRCA1 to DNA [150]. In addition to its UIMs, RAP80 binding to SUMO at DNA damage sites via a SUMO-interacting motif (SIM) is also important for its recruitment to DSB sites [151,152]. Notably, BRCA1 contains a RING domain and forms a stable heterodimer with the RING domain of BARD1 to function as a ubiquitin E3 ligase. While BRCA1/BARD1 has been implicated as the E3 for a range of substrates at DNA damage sites, including histone H2A and CtIP [153], several studies suggest that the ligase activity of BRCA1 is not critical for its tumour suppressive functions and role in promoting HR, although its ability to interact with BARD1 through the RING domain and other proteins in the BRCA1-A complex is important [154–156]. The mechanisms regulating the competition between 53BP1 and BRCA1 are still not well defined, although clearly an antagonistic relationship exists [157–159]. Further studies will help determine the complex relationship between these two proteins as well as the determinants for DSB pathway choice.

### 4.3. Other ubiquitin E3 ligases linked to the DNA damage response

A number of other ubiquitin E3 ligases have been implicated in the DDR. While negative regulation of ubiquitylation signalling events is a well-documented function of DUBs [160], RNF169 has been found to add an additional layer of regulation to the early ubiquitylation signalling cascade initiated by RNF8/168, by also acting as a negative regulator of the pathway. RNF169 is closely related to RNF168, with a very similar overall domain architecture [137]. Recent studies have shown that RNF169 competes with 53BP1 and BRCA1/RAP80 binding to RNF168-ubiquitylated histone H2A and consequently affects the balance of repair between HR and NHEJ [137,161,162]. In addition, the two HECT-E3 ligases TRIP12 (thyroid hormone receptor interactor 12) and UBR5 (ubiquitin protein ligase E3 component amino-recognin 5) function to limit RNF168 and ubiquitin accumulation at DSB sites by regulating steady-state levels of RNF168 [163]. Another ubiquitin E3, the RNF20/RNF40 heterodimer, is responsible for histone H2B mono-ubiquitylation on transcriptionally active chromatin and has also been shown to cause histone H2B K120 mono-ubiquitylation following DNA damage, which appears important for timely repair by both HR and NHEJ [164,165]. FANCL is the RING E3 subunit of the Fanconi anaemia complex [166], which functions with the E2 UBE2T [167] to mono-ubiquitylate FANCD2 and FANCI following interstrand cross-link (ICL) detection, and is therefore crucial for ICL repair. FANCD2/FANCI mono-ubiquitylation promotes retention of the core Fanconi anaemia complex on damaged chromatin and has been shown to be an essential step in coordinating recruitment of a number of key proteins to ICL repair sites, including the nuclease FAN1 [168–170] and CtIP [171]. Both PRP19 (pre-mRNA processing factor 19) and RFWD3 (RING finger and WD repeat domain 3) have been implicated in ATR activation following the generation of RPA-coated ssDNA [172–176]. RFWD3 is a RING ubiquitin E3 that has been shown to promote p53 stability following DNA damage [177]. RFWD3 also physically interacts with RPA and appears important for DNA DSB repair [175,176]. PRP19 is a U-box ubiquitin E3, which in a complex with CDC5L, PRL1 and SPF27 has an established role in pre-mRNA splicing [178]. Recent studies have shown that PRP19 also interacts with RPA and suggest that PRP19 is important for the ubiquitylation of RPA following DNA damage, which promotes ATRIP accumulation and subsequent ATR activation at damaged sites [173,174]. How the functions of PRP19 and RFWD3 are coordinated in respect to DNA damage is not currently known.

STUBLs (SUMO-targeted ubiquitin ligases) are SIM (SUMO-interacting motif)-containing ubiquitin E3 ligases that bind and ubiquitylate sumoylated proteins. They play important roles in the dissociation of highly sumoylated complexes at DSB sites and link sumoylation to proteasomal degradation. The first described STUBL was Slx8-Rfp in *Schizosaccharomyces pombe* (Slx5–Slx8 in *Saccharomyces cerevisiae*), the lack of which was shown to cause an accumulation of sumoylated proteins and yeast DNA damage hypersensitivity [179]. A similar level of DNA damage sensitivity is seen in human cells deficient in the Slx5–Slx8 homologue RNF4, which is important for DSB repair by both HR and NHEJ as well as for the release/turnover of several key DSB repair proteins from DNA damage sites, including MDC1, 53BP1, RPA, RAD51, FANCI and FANCD [180–182]. RNF4 accumulation at DNA DSB sites is dependent on its tandem SIM domains, and its recruitment most probably occurs in response to a collection of sumoylated proteins at DNA damage sites, where it functions to ubiquitylate and target proteins for removal via the proteasome and/or the p97/VCP segregase [181,182]. In fact, it has recently been demonstrated that the ubiquitin E3 ligase activity of RNF4 depends on RNF4 binding to SUMO2/3 polymeric chains and subsequent RNF4 dimerization [183]. In addition to its role in promoting the turnover of proteins, RNF4 might also be important for the formation of hybrid SUMO/ubiquitin chains at DNA damage sites, which mediate the recruitment of proteins containing both SIMs and UBDs, such as RAP80 [151]. A second human STUBL with a role in the DDR has now also been described in the literature—RNF111 (also known as Arkadia)—although in this case, the function of ubiquitylation is not to target the substrate for proteasomal degradation; rather, RNF111 preferentially ubiquitylates sumoylated XPC following UV damage to promote its recruitment to damaged DNA [184].

### 4.4. Links between the ubiquitin–proteasome system and VCP/p97 in the DNA damage response

The proteasome is a more than 2.5 megadalton protease that functions to degrade ubiquitylated proteins [185], and consists of two major components: the 28-subunit core particle (also known as the 20S subunit) and the regulatory particle (also known as the 19S subunit). Substrate entry into the central chamber of the barrel-shaped 20S subunit, where the proteolytic active sites reside, is regulated by the 19S subunit, which recognizes ubiquitin tagged proteins. Proteasomal subunits can be detected at DNA DSB sites [181,186] and a functional proteasome is important for DNA DSB repair, being required for the turnover of DNA damage checkpoint proteins such as CDC25A [187] as well as the turnover of DNA repair proteins such as MDC1, BRCA1 and RPA [181,188,189].

VCP (valosin-containing protein; also known as p97 or Cdc48 in budding yeast) is a hexameric AAA (ATPases-associated with various cellular activities)-ATPase, that uses ATP hydrolysis to structurally unfold or remodel proteins [190]. VCP associates with a number of different ubiquitin-binding domain-containing cofactors that regulate the activity and functions of VCP and of which UFD1 and NPL4 are the most studied. The segregase/chaperone functions of VCP are the best described, where VCP-UFD1-NPL4 binds misfolded and/or polyubiquitylated proteins, extracts them from protein complexes, cellular surfaces or chromatin and delivers them to the proteasome for degradation. By its association with ubiquitin chain-editing proteins and DUBs, VCP can also modify ubiquitin chains to facilitate protein turnover. A growing amount of evidence demonstrates that chromatin extraction of DNA repair proteins by VCP is a general mechanism for removing ubiquitylated proteins from DNA damage sites [191]. In global genome nucleotide excision repair (GG-NER), there is strong evidence to suggest that VCP is important for the removal of CRL4^DDB1^-ubiquitylated DDB2 and XPC from DNA damage sites [192], as well as for the extraction of chromatin/PCNA-associated CDT1 following DNA damage [193]. VCP is also important for DSB repair and is recruited to DNA DSB sites, where it most probably functions to facilitate the clearance of K48-linked ubiquitylated proteins [191,194]. DVC1 (also known as SPRTN and C1orf124) has recently been shown to be a VCP adaptor protein, required for the recruitment of VCP to stalled replication forks [195,196]. DVC1 depletion in cells enhances UV-induced mutagenesis, and it has been proposed that VCP might facilitate extraction of translesion synthesis polymerases during post-replication repair [195,196]. *In vivo*, DVC1 deficiency causes genomic instability and progerioid phenotypes [197,198]. In addition, patients with biallelic germline mutations in DVC1 develop early onset hepatocellular carcinoma [198].

Interestingly, UFD1 and NPL4 also bind SUMO through their SIMs [199], and in budding yeast have been shown to extract sumoylated Rad52 from chromatin, thereby restricting Rad51-dependent HR [200]. The function of VCP may therefore extend beyond the ubiquitylation system and most probably plays critical but as-yet relatively undefined roles in DSB repair.

### 4.5. Deubiquitylating enzymes and double-strand break repair

Various DUBs have now been directly implicated in DNA DSB repair, and the role of DUBs in the DDR has been more extensively reviewed elsewhere [160]. OTUB1 promotes p53 stabilization and also functions as a negative regulator of RNF168-dependent ubiquitylation at sites of DNA damage [201,202]. OTUB1 inhibits the RNF168 E2 enzyme UBE2N (and probably also other E2s) independently of its de-ubiquitylating activity [203,204]. USP44 is recruited to sites of DNA damage and has been shown to antagonize RNF168 recruitment to such sites [205]. It is also important for the regulation of the mitotic spindle checkpoint [206,207]. USP1 functions in DNA ICL repair by regulating FANCD2 mono-ubiquitylation [208], and in its absence cells are hypersensitive to DNA cross-linking agents because of constitutive chromatin association of FANCD2 [209]. USP28 is recruited to DNA DSBs in a 53BP1-dependent manner and was reported to be important for the stability of NBS1, CHK2, 53BP1, CLSPN and TOPBP1 after DNA damage [210]. It has been subsequently demonstrated, however, that USP28 depletion has little effect on DNA repair, and therefore USP28 is unlikely to play a major role in regulating DNA DSB responses [211]. POH1 (also known as PSMD14) is a proteasome associated DUB which has been shown to negatively regulate K63 ubiquitin and 53BP1 accumulation at DNA DSBs as well as to facilitate HR through promoting RAD51 loading [186]. BAP1 (BRCA1-associated protein 1; also known as UCHL2) is encoded by a tumour suppressor gene, which is frequently mutated in a range of cancers [212]. Depletion of BAP1 results in hypersensitivity to DNA damaging agents, and it has been proposed that BAP1 is important for the recruitment of HR effector proteins and the polycomb deubiquitylase complex to sites of DNA damage [213,214]. In addition to the above, a recently published siRNA screen has systematically characterized all known human DUBs against a panel of DDR assays, thereby highlighting likely DSB repair functions for various DUBs [215]. One of the top hits from this screen, UCHL5, was subsequently shown to promote DNA resection and HR through stabilization of NFRKB (nuclear factor related to kappa-B-binding protein), a component of the INO80 chromatin-remodelling complex [215].

## 5. Post-translational modification with NEDD8

**Figure 3.**
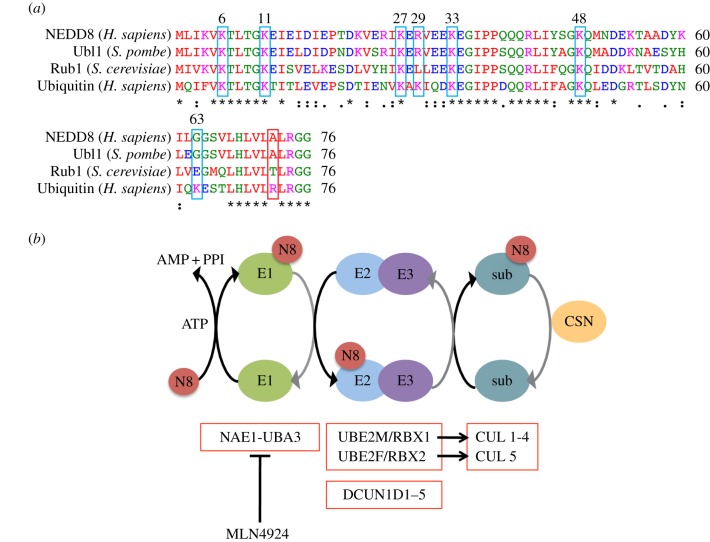
NEDD8 sequence homology and the neddylation cascade. (*a*) Sequence alignment of human NEDD8, ubiquitin and the NEDD8 homologues in *Saccharomyces pombe* (ubl1) and *Saccharomyces cerevisiae* (rub1) using ClustalW2 (http://www.ebi.ac.uk/Tools/msa/clustalw2/). Lysines critical for ubiquitin chain formation are outlined in blue. Residue 72 (Arg in ubiquitin and Ala in NEDD8) critical for E1 specificity is outlined in red. Colours of amino acids relate to their physiochemical properties: red, small (small + hydrophobic, including aromatic Y); blue, acidic; magenta, basic; green, hydroxyl + sulfhydryl + amine + G. Asterisk (*) denotes fully conserved residue, the symbols :/. denote conservation between groups of strongly/weakly similar properties. (*b*) Representation of the major neddylation pathway components. NEDD8 (N8) is conjugated in an ATP-dependent cascade involving an E1 (NAE1-UBA3), E2 (UBE2M or F) and E3 (RBX1 or -2) to cullin substrates (sub). The enzymatic pathway is analogous to ubiquitylation (see figure 2 for details). DCUN1D1–5 are cofactors for the NEDD8 E3s. Neddylation is reversed by the CSN complex. MLN4924 inhibits UBA3. See text for details.

In 1992, 10 genes were identified that showed developmental downregulation of their expression in the mouse brain and were named NEDD1–10 (neural precursor cell expressed, developmentally downregulated 1–10) [216]. NEDD8 was later shown to be a UBL, and of the UBLs that is the most highly related to ubiquitin at the sequence and secondary structure levels (figure 3*a*) [217,218]. NEDD8 is conjugated to target proteins by an enzymatic neddylation cascade that is extremely well conserved from yeast to human, and is an essential pathway in all organisms studied, with the curious and currently unexplained exception of *Saccharomyces cerevisiae* [219,220].

Like ubiquitin, NEDD8 is first synthesized as a precursor (from a single *NEDD8* gene however) that is processed at the conserved carboxyl-terminal Gly76 residue by the hydrolase activity of deneddylating enzymes, exposing a di-glycine motif that is required for NEDD8 covalent attachment to target substrates. Both NEDP1 (also known as DEN1 or SENP8) and UCHL3 have been described as NEDD8 processing enzymes [221,222]. NEDD8 is conjugated to a substrate lysine (predominantly on cullin proteins) in a three-step enzymatic process analogous to ubiquitylation, using E1, E2 and E3 enzymes specific for neddylation (figure 3*b*). The NEDD8 E1, NAE (NEDD8 activating enzyme), is composed of the NAE1-UBA3 heterodimer, with NAE1 and UBA3 being homologues of the amino- and carboxyl-terminal regions, respectively, of the ubiquitin E1 UBE1. Given the extent of homology between both NEDD8 and ubiquitin, as well as between NAE and UBE1, an interesting question is how specificity between the UBL and E1 is achieved. Site-directed mutagenesis studies identified Arg72 in ubiquitin (Ala72 in NEDD8) as being critical for E1 specificity [223], as Arg190 on UBA3 repels Arg72 on ubiquitin [224]. A NEDD8 Ala72Arg mutant and ubiquitin Arg72Ala mutant is capable of binding the ubiquitin and NEDD8 E1, respectively [218,225,226], highlighting the importance of this residue. Specificity between the respective ubiquitin and NEDD8 pathway enzymes is maintained through a unique interaction between an amino-terminal sequence on UBE2M and a binding groove of UBA3 [227]. Despite these features, however, NEDD8 activation by the ubiquitin E1 UBE1 is possible and has been demonstrated in circumstances when the NEDD8:ubiquitin balance in cells is perturbed—either through overexpression of NEDD8 or following chemical or physiological depletion of ubiquitin [228,229].

There are two known NEDD8 E2s: UBE2M and UBE2F [219,230–232], which preferentially partner with the E3 RBX1 (also known as ROC1) or RBX2 (also known as ROC2 or RNF7), respectively [232]. RBX1 and -2 are RING E3s which simultaneously bind their substrate and NEDD8∼UBE2M/F to catalyse NEDD8 transfer [233]. Interestingly, RBX1 and RBX2 also function as the ubiquitin E3s for cullin-RING complexes [234,235]. An additional group of proteins—Dcn1 (defective in cullin neddylation 1) in budding yeast and DCUN1D1–5 (defective in cullin neddylation 1, domain-containing 1–5, also known as DCNL1–5) in humans—have been described that act as cofactors for RBX1 and RBX2, potentiating their neddylation activity [233,236–240]. The DCUN1D proteins contain a UBD and a conserved, unique PONY (potentiating neddylation) domain that binds amino-terminally acetylated UBE2M/F and the cullin substrate [238,240]. DCUN1D1–5 do not appear to affect E2/E3 substrate specificity and are each able to bind all of the cullins via their PONY domains. However, they potentiate the neddylation activity of different E3-cullin complexes to varying degrees [240]. Currently, substrate specificity of NEDD8 is only loosely defined at the level of the E2, with UBE2M/RBX1 and UBE2F/RBX2 preferentially neddylating CUL1–4 or CUL5, respectively [232]. Why five human orthologues of DCN1 exist remains unknown, and subcellular localization or varying expression levels of the DCUN1D proteins may prove to be important for spatio-temporal regulation of cullin-RING ligase (CRL) activity [239–241].

Like ubiquitin, NEDD8 also contains internal lysine residues suitable for chain formation, although, at least in yeast, these conserved residues do not appear to be functionally essential [242]. Although NEDD8 chain formation has been reported in the literature, *in vivo* it has only been described on non-cullin substrates when NEDD8 is overexpressed, so the physiological relevance of NEDD8 chains remains unknown [228,243,244].

As for other protein PTMs, neddylation is a reversible process, with the CSN (COP9 signalosome) being the predominant de-neddylase. The CSN is a conserved complex of eight proteins (CSN1–8), named after a group of COP (constitutive photomorphogenesis) mutants that showed constitutive photomorphogenesis, pigmented seed coats and premature death in plants [245]. The JAMM motif (Jab1/MPN domain) of CSN5 was later shown to be critical for the de-neddylating activity of the CSN and linked the CSN to the regulation of the CRLs [246]. A complete CSN complex is required for full enzymatic activity *in vitro* [247], with CSN5 being constitutively inactive until binding of the CSN to the cullin substrate triggers conformational changes that result in activation of isopeptidase activity [248]. The CSN is the only isopeptidase that efficiently cleaves NEDD8 from cullin substrates *in vivo*, although a number of other proteins have been shown to possess NEDD8 isopeptidase activity *in vitro* [249].

### 5.1. Neddylation substrates: the cullins

#### 5.1.1. Cullin-RING ligases

**Figure 4.**
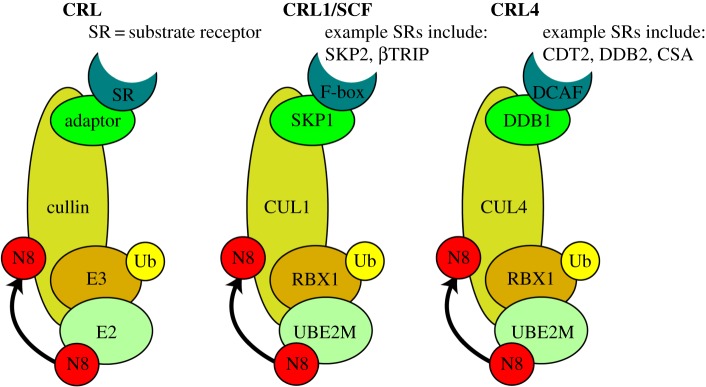
Cullin-RING ligases (CRLs). Simplified diagram of CRL structure. The cullin forms the backbone of the CRL complex. Cullin homology domains at the carboxyl-terminus are required for binding to the E3 and the amino-terminus interacts with the substrate adaptor proteins where required (there is no known substrate adaptor for CUL3) [242]. Neddylation of the cullins on a conserved carboxyl-terminal lysine induces conformational changes to promote ubiquitylation of CRL substrates. SKP1 and DDB1 are the substrate adaptor proteins for CRL1 and CRL4 complexes, respectively. F-box proteins and DCAFs are the substrate receptors for CRL1 and CRL4 complexes, respectively. See text for more details.

The cullins are the best-defined NEDD8 substrates, of which there are eight encoded by the human genome (CUL1, 2, 3, 4A, 4B, 5, 7 and PARC), and they all share an evolutionarily conserved cullin homology domain and carboxyl-terminal neddylation site. The APC2 subunit of the anaphase promoting complex/cyclosome (APC/C) also contains cullin homology domains, but lacks the carboxyl-terminal NEDD8 consensus sequence (IVRIMKMR) and is therefore functionally distinct from the other cullins. Cullins are molecular scaffolds for the CRLs, which are E3 ubiquitin ligases responsible for up to 20% of all ubiquitin-mediated proteasomal degradation [250–252]. CUL1-containing CRL1 is the archetypal CRL and is also commonly known as SCF (SKP, cullin, F-box containing complex). The CRLs contain three major components: a cullin scaffold, a RING finger protein (RBX1 or RBX2; that recruits a ubiquitin-charged E2 enzyme, as well as the NEDD8 loaded E2) and a substrate recognition protein, that may or may not be associated with a substrate adaptor protein, which places substrates in proximity to the ubiquitin E2 enzyme to facilitate ubiquitin transfer (figure 4). Cdc34 is the only known E2 to associate with CRLs in yeast, although both CDC34a/b (also known as UBE2R1 and UBE2R2) and UBCH5 (also known as UBE2D1) have been described as ubiquitin E2s for the CRLs in humans, and others may also exist [250,253].

SKP1 (S-phase kinase-associated protein 1) and DDB1 (damage-specific DNA-binding protein 1) are the best-described substrate adaptor proteins and link CUL1 and CUL4A/B, respectively, to their substrate receptors. The substrate receptors for CUL1, 2, 3, 5 and 7 are structurally related FBX (F-box-like) proteins, although CUL4A and CUL4B use distinct substrate receptors known as DCAFs (DDB1- and CUL4-associated factors). Many of the DCAFs contain WD40-like repeats [254] of which CDT2 (chromatin licensing and DNA replication factor 2) and DDB2 (damage-specific DNA-binding protein 2) are examples. The WD40 domain is one of the most abundant domains encoded by the eukaryotic genome. They commonly mediate protein–protein interactions, and many WD40 domain-containing proteins have been found as interactors of CUL4A and CUL4B in proteomic studies [255,256]. A single substrate receptor can bind multiple substrates however. For example, CRL4^CDT2^ binds CDT1, p21 and SETD8, among others. Therefore, the substrate specificity of the CRLs depends on both the combination of its components and also the cellular context of the substrate itself; for example, CRL4^CDT2^ only binds CDT1 when it is associated with chromatin-bound PCNA (see later sections for more details). Proteomics studies have revealed the complexity of the CRL network [255] and diversity of CRL substrates [257,258], culminating in more than 200 unique CRL complexes that play key roles in cell cycle regulation, embryogenesis, DNA replication and repair [251].

#### 5.1.2. Regulation of cullin-RING ligase activity

CRL activity towards its substrate is tightly controlled, and regulation is multi-factorial. Neddylation of the cullins, on a conserved carboxyl-terminal lysine, serves to increase the ubiquitylating activity of the CRLs by triggering conformational changes within the cullin and by preventing association with the CRL inhibitor CAND1 (cullin-associated NEDD8-dissociated 1) [259]. CAND1 binds to de-neddylated cullins, promotes dissociation of the cullin from the substrate receptor and inhibits CRL activity *in vitro* [260,261]. As well as showing inhibitory CRL effects, however, genetic studies demonstrated that CAND1 also promotes CRL activity, with this functional paradox being explained by the importance of CAND1 for maintaining an available pool of substrate receptors and therefore promoting the dynamics of CRL formation and dissociation [262–264].

CSN regulation of CRL activity is also more complex than initially thought. CSN association with the CRL maintains the cullin in a de-neddylated state. This, along with its association with the de-ubiquitylating enzyme USP15 [265], inhibits the ubiquitylation of CRL substrates and protects substrate receptor proteins from auto-degradation by the CRL. In some cases, the CSN may also physically block substrate access. Taken together, CSN dissociation from the CRL has therefore become a recognized mechanism for promoting substrate ubiquitylation [266–269].

Independently of cullin association with the negative regulators CSN and CAND1, cullin neddylation and thus activation also appears to be dependent on the formation of a complete CRL unit, including cullin binding to adaptor proteins and substrate receptor subunits, possibly through inducing a conformational change or dimerization of the CRL, which promotes neddylation [270]. PTMs of the substrate binding motif or ‘degron’ also regulate CRL interaction with its target and subsequent activation in some cases [271]. Further structural and biochemical studies will better determine the mechanisms of CRL activation. Clearly, however, spatio-temporal regulation of CRL activity is critical and is achieved through a fine balance of promoting and inhibitory mechanisms.

### 5.2. The neddylation inhibitor MLN4924

Many CRL components are either overexpressed or mutated in human cancers [272,273], making CRL inhibition an attractive anti-cancer strategy. In addition, success of the proteasome inhibitor bortezomib (Velcade^®^; Takeda Oncology) for the treatment of multiple myeloma and mantle cell lymphoma has paved the way for novel therapeutics that inhibit ubiquitin-mediated protein degradation pathways.

MLN4924 (Takeda Oncology) is an irreversible inhibitor of UBA3 that potently and specifically inhibits UBA3 activation of NEDD8 in cells, and subsequently inhibits global NEDD8 conjugation and CRL activity [252]. MLN4924 is an AMP analogue which is catalysed by UBA3 to form a covalent UBA3-MLN4924 adduct that can no longer be used by the NEDD8 machinery [274]. Therefore, treatment of cells with MLN4924 rapidly leads to the accumulation of CRL substrates, including CDT1, p27 and NRF2, among others [252]. The predominant cellular phenotype, in a range of cell lines tested, following prolonged (8 h) MLN4924 treatment is S-phase arrest and DNA re-replication, thought to be largely due to CRL4^CDT2^ inhibition [275], although CRL1 is also likely to contribute. CRL4^CDT2^ inhibition causes stabilization of CDT1 and SETD8 (also known as SET8 or PR-SET7), both of which contribute to the re-replication observed. CDT1 is required for loading of the DNA replication helicase complex (MCM2–7) onto chromatin in the G1 phase of the cell cycle, and CRL4^CDT2^/SCF^SKP2^-mediated degradation of CDT1 during S-phase prevents DNA re-replication [276]. SETD8 catalyses histone monomethylation on Lys 20 of histone H4 (H4K20me1), and PCNA-dependent degradation of SETD8 by CRL4^CDT2^ during S-phase inhibits DNA re-replication (although the mechanism by which SETD8 promotes loading of the replication complex onto chromatin remains unknown) [277,278]. DNA re-replication is a recognized cause of DNA damage [279] and prolonged MLN4924 treatment causes replication-dependent activation of ATM- and ATR-mediated DDR signalling, DSB formation as measured by γH2AX formation and eventual apoptosis or cellular senescence [279–281]. In addition to re-replication itself being a cause of DNA damage, recently it has been shown that CRL-dependent ubiquitylation is required for dissociation of the DNA replisome in *Saccharomyces cerevisiae* and *Xenopus* [282,283]*.* It will be interesting to determine how failure to remove the MCM complex on completion of DNA replication might contribute to DSB generation following treatment with MLN4924. Regardless of mechanism, cell survival in yeast mutants that undergo DNA re-replication or in human cells following MLN4924 treatment is reduced in DSB repair deficient backgrounds [284,285], clearly suggesting that cell death following MLN4924 treatment is at least in part due to the generation of DNA DSBs.

Pre-clinical data suggest that inhibiting neddylation might be an effective anti-cancer strategy for solid tumours and haematological malignancies [286]. MLN4924 is well tolerated and has shown anti-tumour activity in phase I oncology trials involving haematological and solid tumours [287] (see also http://meetinglibrary.asco.org). Phase I combination trials are on-going (http://www.cancer.gov/clinicaltrials).

### 5.3. Non-cullin neddylation substrates

A number of non-cullin substrates for NEDD8 have been described in the literature, including p53 [288], the ribosomal protein L11 [289] and histone H4 [290]. In addition, proteomics studies have identified neddylation targets both within and outside the known CRL network [243,244]. However, these studies have largely relied on the overexpression of tagged-NEDD8 constructs as their bait, and as conjugation of NEDD8 to target proteins using the ubiquitin machinery can occur in these circumstances [229], it is difficult to be convinced that proteins other than cullins are true NEDD8 substrates *in vivo*. It is also worth noting that, while MLN4924 specifically inhibits neddylation, it also leads to potent inhibition of CRL ubiquitylating activity. Consequently, demonstrating that neddylation of a substrate is MLN4924 dependent does not discriminate between conjugation being mediated by the NEDD8 or ubiquitin machineries. Furthermore, histones are heavily ubiquitylated by CRLs and are often identified as neddylation substrates when NEDD8 is overexpressed. A recent excellent review by leading laboratories in the neddylation field has suggested criteria for identifying physiological neddylation substrates and, to date, only the cullins fulfil all these criteria [291].

## 6. Role of neddylation in the DNA damage response

### 6.1. CUL4A and CUL4B

Of all the cullins, CUL4A and CUL4B are the most strongly linked to the DDR. Higher eukaryotes such as *Homo sapiens*, *Mus musculus* and *Xenopus tropicalis* have both types of CUL4, which share approximately 85% sequence identity and are derived from a common CUL4 ancestor found in *Caenorhabditis elegans*, *Drosophila melanogaster* and *S. pombe*. At a cellular level, a large amount of functional redundancy exists between CUL4A and CUL4B. Some non-overlapping functions of the two proteins have been revealed by mouse genetic studies however, because although *Cul4A^−/−^* mice are viable with no overt developmental defects [292], presumably due to functional compensation by *Cul4B*, *Cul4A^−/−^* male mice are sterile, which is thought to be due to a failure to resolve recombination crossover intermediates during meiosis [293]. In addition, mutations in CUL4B are linked to an X-linked mental retardation syndrome in man [294] and *Cul4B^−/−^* mice display embryonic lethality due to developmental failure of extra-embryonic tissues [295]. Beyond these limited examples, CUL4A and CUL4B appear to be functionally redundant with one another, and the roles described below for CRL4 in the DDR cannot be linked to either one of the proteins in isolation.

### 6.2. CRL4 and nucleotide excision repair

Both GG-NER and TC-NER (table 1) use mechanisms involving CRL4 to recognize DNA lesions and promote repair [88]. DDB2 is the DNA damage sensor protein for GG-NER and is the substrate receptor of the CUL4–DDB1 CRL complex (CRL4^DDB2^), which in the nuclear fraction of unperturbed cells is closely associated with the CSN [296]. In the current model, upon binding to UV-damaged sites, CRL4^DDB2^ dissociates from the CSN, leading to activation of the CRL and ubiquitylation of XPC and DDB2 [266,296]. Interestingly, the inhibitory effects of the CSN on CRL4^DDB2^ appear to be independent of CSN5 de-neddylase activity [266]. Dimerization of CRL4^DDB2^ upon binding to DNA may also be important for facilitating ubiquitylation of its substrates [297]. Ubiquitylation of DDB2 reduces its affinity for DNA and causes its proteasome-mediated degradation, whereas ubiquitylation of XPC potentiates its association with the DNA, enabling repair [298]. In addition, CRL4^DDB2^ ubiquitylates histone H3 and H4 in the vicinity of the DNA lesions, facilitating access of the NER repair machinery [299]. Mutations in the DNA-binding region of DDB2 give rise to the archetypal NER syndrome xeroderma pigmentosum [300,301].

CSA (Cockayne syndrome type A protein) is another substrate receptor for CUL4–DDB1, and CRL4^CSA^ is recruited to TC-NER sites where it is believed to ubiquitylate the SWI/SNF ATPase CSB (Cockayne syndrome type B protein) and is important for repair and ensuing transcriptional re-start [266,302]. Although CSA and DDB2 are quite distinct at the sequence level, they are structurally very similar in their complex formation with DDB1, and like CRL4^DDB2^, CRL4^CSA^ activity appears to be regulated by its association/dissociation with the CSN [266].

### 6.3. Cullin-RING ligase regulation of DNA damage cell cycle checkpoint responses

CRLs play prominent roles in cell cycle regulation [276] and are also important for cell cycle checkpoint responses following DNA damage. As described above, CDT1 degradation by CRL4^CDT2^/SCF^SKP2^ during normal S-phase is essential in preventing DNA re-replication. In addition, following DNA damage, CRL4^CDT2^ promotes CDT1 degradation in a chromatin-bound PCNA-dependent manner. CDT1 contains a ‘PIP degron’, which is made up of a canonical PCNA binding motif (the PIP box; through which CDT1 binds PCNA) and a basic patch of four amino acids downstream (B+4), which mediates the interaction between CDT1 and CRL4^CDT2^. CRL4^CDT2^ interacting with both PCNA and CDT1 via the PIP degron is a common mechanism by which CRL4^CDT2^ interacts with its substrates [303], although how this interaction is regulated to occur only in the context of chromatin-bound PCNA is not known [304].

CDC25A is a phosphatase required for activation of cyclin-dependent kinases, enabling cell cycle progression. Following DNA damage, CDC25A is phosphorylated in a predominantly CHEK1/CHEK2-dependent manner, leading to its ubiquitylation by SCF^βTRCP^, degradation and subsequent cell cycle arrest [305]. Similarly, a phospho-degron signal on CLASPIN (a protein required for ATR-mediated CHEK1 activation following stalled replication forks and DNA end resection during HR) triggers its ubiquitylation by SCF^βTRCP^ and subsequent degradation, enabling checkpoint recovery [306,307].

### 6.4. Neddylation and double-strand break repair

*In vitro* studies using *Xenopus laevis* egg extracts have shown that, upon binding DNA, Ku80 is polyubiquitylated with K48 chains by the cullin 1 containing complex SCF^FBXL12^ and removed from the DNA [308,309]. Truncation mutants of Ku80 that are proficient in DNA binding, but not DNA repair, are efficiently ubiquitylated, suggesting that in this system DNA binding rather than the completion of repair is the signal for Ku ubiquitylation and release [309]. The mechanism by which Ku is extracted from the DNA remains unclear, however. Postow *et al*. [309] showed that Ku removal is independent of the proteasome and hypothesized that VCP unfolds Ku, thereby promoting its removal from DNA [310]. Ku forms a highly stable ring-like structure that threads onto DNA ends [311], and failure to remove Ku from repaired DNA will likely interfere with cellular processes such as DNA replication and transcription [312,313], highlighting the need for an active mechanism to release DNA bound Ku [310]. It has not yet been determined whether a CRL-dependent mechanism of Ku release is conserved in human cells.

In human cells, inhibition of NEDD8 conjugation has been shown to hypersensitize cells to DNA damaging agents such as mitomycin C, cisplatin and IR [314–321]. Synergy between MLN4924 and DNA cross-linking agents also clearly exists across a range of cell lines [314,316–319]. The mechanisms behind this synergy are not fully elucidated and have not been consistent between studies [316] but may include inhibitory effects on assembly of the Fanconi complex [317], promotion of reactive oxygen species (ROS) generation [318] and increased replication fork collapse due to BRCA2/RAD51 stabilization [314]. Given the wide-ranging cellular effects of neddylation inhibition, it is likely that the synergy observed is multi-factorial and potentially cell-type specific. Further studies will refine our understanding of the mechanisms involved.

Recent studies have linked neddylation with DSB repair more directly and NEDD8 has been shown to localize to DNA damage sites [290]. In this study by Ma *et al.*, it was concluded that RNF111 functions as the NEDD8 E3 ligase responsible for NEDD8 accumulation at sites of DNA damage. More specifically, the authors concluded that RNF111-mediated neddylation of histone H4 is essential for RNF168 recruitment to DSB sites [290]. RNF111, also known as Arkadia, is a ubiquitin E3 ligase with an established role in TGF-β signalling [322], and more recently has been shown to function as a STUBL in PML degradation and in NER [184,323]. Whether the effects of RNF111 depletion on DSB repair reported by Ma *et al.* are due to the ubiquitylation rather than the suggested neddylation activity of RNF111 has not been established. In addition, as recently published, like most ubiquitin E3s, the RING structure of RNF111 does not favour interaction with the NEDD8 E2 UBE2M [291,324], and as a consequence, it would be interesting to determine the NEDD8 E2 responsible for the proposed neddylation function of RNF111.

Other studies linking neddylation and DSB repair include a published report by Wu *et al*. [325] that the CUL1 substrate receptor SKP2 is important for NBS1 K63 ubiquitylation and subsequent ATM activation. Interestingly, it has also been demonstrated that NBS1 ubiquitylation by RNF8 is important for efficient NBS1 recruitment to DNA damage sites [326], and both pathways appear to be important for repair by HR. More recently, it has been proposed that RNF111-mediated neddylation regulates the extent of DNA end resection and neddylation inhibition increases rates of HR [327]. RNF168 has also been reported to function as a NEDD8 E3 ligase in response to DNA damage, where it has been reported to neddylate histone H2A and subsequently negatively regulate the ubiquitylation of H2A and the downstream recruitment of BRCA1 [328]. As discussed above, the existence of non-cullin neddylation substrates is a contentious issue in the field [291], and further studies are required to substantiate H2A as a neddylation substrate and RNF168 as a NEDD8 E3 ligase.

## 7. Summary

The importance of PTMs in the DDR has been appreciated for many years, and the role of ubiquitin and the UBLs in DSB repair has been an area of active research over the last decade. A major unanswered question in the field is the identity of the RNF8 substrate responsible for RNF168 recruitment. In addition, it is likely that the full spectrum of ubiquitin-system proteins important for the DDR has not yet been fully established. Indeed, it seems likely that further work in this area will uncover new mechanistic insights into DDR processes as well as provide promising new drug targets.

Given the complexity of the crosstalk between SUMO and ubiquitin systems during cellular responses to DNA DSBs [329], it would probably not be surprising if similar levels of crosstalk exist between the neddylation and ubiquitylation systems. As the predominant function of neddylation in the literature to date is to regulate the ubiquitylating activity of the CRLs, we feel that it will be particularly interesting to determine how CRL activity is regulated in the context of responses to DNA damage. While MLN4924 is an extremely useful tool for investigating the cellular consequences of neddylation inhibition, prolonged treatment with MLN4924 has wide-ranging cellular effects and generates high levels of DNA DSBs [252]. Potent inhibition of neddylation is achieved within minutes in cells treated with MLN4924, so short treatment times should be used wherever possible and appropriate experimentally. Likewise, given the potential utilization of NEDD8 by the ubiquitin machinery, caution should be exercised when interpreting results using overexpressed NEDD8 systems, and reagents should be validated as being specific for NEDD8. Inhibitors specific for individual CRL complexes are not available, but would be extremely helpful for determining the fine-tuning of the complex CRL network.

Studying the mechanisms by which ubiquitin and the UBLs regulate DSB repair and associated processes is a technical challenge, not least because of the functional redundancy in these systems. A better understanding of how these PTMs interconnect, as well as the development of more small molecule inhibitors such as MLN4924 will enable the cellular functions of these and connected enzymatic pathways to be determined through approaches that until now have not been feasible. In particular, further advances in proteomics techniques will better define the substrates of various PTMs, and evolving gene-targeting and cell engineering approaches [330,331] will complement the use of inhibitors in determining the functions of PTM pathway members. Being frequently mutated or overexpressed in human cancers, the ubiquitin and UBL pathways offer a spectrum of attractive drug targets, and with the recent approval of PARP inhibitor olaparib/Lynparza^TM^ (AstraZeneca) by the European Medicines Agency and the US Food and Drug Administration for the treatment of BRCA-mutated, high-grade serous epithelial ovarian cancer, the precedent is now set for DDR inhibitors in the clinic.
